# Academy of Stomatology

**Published:** 1898-11

**Authors:** 

**Affiliations:** Academy of Stomatology


### ACADEMY OF STOMATOLOGY.

The regular monthly meeting of the Academy of Stomatology was held at the rooms of the Academy on the evening of June 28, 1898, the President, Dr. M. H. Cryer, in the chair.

After the transaction of routine business, "Incidents of Practice" with discussions were presented by the Fellows of the Academy.

### INCIDENTS OF PRACTICE.

*The President.*—As there is no special paper for this evening, "Incidents of Practice" will be in order. If any one has any interesting subject of office practice to present, we will now listen to him.

*Dr. Huey.*—While some one is making up his mind to tell us something of interest, I would like to ask if any one knows of a paper tooth that has been invented and placed on the market. I saw an account of it in a local paper a few days ago. The article said that for durability it excelled the porcelain tooth; that it was easy of adjustment, and made a very natural substitute. I do not know anything about it.

*Dr. William Trueman.*—I saw a statement concerning it in one of the journals quite recently, and thought it was a canard; I did not think it was a practical thing. In connection with this, I remember seeing in a dry-goods store on Eighth Street some years ago full sets of teeth, upper and lower, made of paper pulp.

*The President.*—As this subject has not interested the Academy very much, we will pass it. I should like to hear from some other members. Gentlemen, we are here to talk. You assemble, two or three together, and talk about the little things that happen in your offices, and we would like to have these cases reported and discussed for the benefit of all.

*Dr. Huey.*—We are all more or less interested in the "moss-fibre" gold recently introduced by the S. S. White Dental Manufacturing Company. I have used something like half an ounce of it, and while I have not yet gained sufficient courage to attempt a restoration, I have found it to work admirably in difficult, inaccessible cavities, especially in repairing gold fillings where recurrence of decay has taken place. I find that I can pack that gold against old fillings very much better than any other form of gold I have tried. I do not know that it will take the place of foil, but I am experimenting with it, and am satisfied with the result of the experiment. With the use of very fine points I find it makes a fairly condensed filling and burnishes down well to the edges of the cavity. I have noticed that it absorbs moisture more rapidly than any other gold, consequently it should be reannealed after any lapse of time or for any subsequent fillings.

*Dr. Roberts.*—I have been using moss-fibre gold quite extensively lately, and it works very nicely. It does not crumble readily. Of course, all fibrous gold and sponge gold will crumble, but there is less waste with moss-fibre gold than with any I have used. I have not used it in contouring incisors, but in molars and bicuspids I have had satisfaction with it. It is soft, and the only fear that I have about its use is that it will work too easily. By using too much of it in a mass one may not get the gold thoroughly condensed, but by using not too large points it works as nicely as any gold. It is very cohesive, and can be used in almost any place, either with special instruments or without. It is easier to work with the special instruments on account of the shape of the instrument alone. Nearly all the instruments are of a ball shape and serrated, not only on the end but on the round sides, with view to the rotation of the instrument in the act of condensing. I have used it with the ordinary corkscrew hand-instruments, with the automatic and engine mallet, and it packs beautifully.

Dr. Gardiner has been using it largely with the electric mallet, and he will tell us his impression of its action under that instrument. It is well to have as large an instrument as will conveniently enter the undercut of cavities, and carry the gold solidly into them. I anneal, then tear off a small portion with pliers and place it in the cavity. I pack almost entirely by hand-pressure, but in contouring I finish up with the automatic or engine mallet.

*Dr. Huey.*—After various experiments, I have found that in annealing over a flame I was apt to burn the edges of small pieces, so I went back to my mica sheath, and I have more satisfaction and less waste. I can get any heat I desire in that way, but usually anneal to a red color.

I find that I can keep my filling very much smoother and prevent pits by first condensing by hand-pressure and then consolidating with the electric mallet.

*Dr. Gardiner.*—I have been using moss-fibre gold with increasing satisfaction for some time, and I like it very much. I first anneal a large piece on mica, and from this tear small portions with pliers to be used without further annealing. In starting a large filling it is much easier to first bridge across the cavity by hand-pressure. After that it can be either worked down by hand-pressure or introduced and worked with the mallet. I work it more rapidly with the electric mallet. In large cavities it promises to be a great time-saver. The danger will lie in the temptation to work it too rapidly, and in that way produce defective work. It should be worked as carefully as any other gold, though it packs much more readily, and in larger pieces. It will be of more use in large cavities than in small ones because of its bulky nature. With a little practice one can use almost any point with it, but I think that shallow serrations are much better. Of course, in beginning a filling the larger points are better, as they prevent rocking. The bottom of the cavity should be as flat as possible, as a convenience in starting. In all cavities that are not very deep the plane of the cavity can easily be made flat without destroying too much tooth-substance. I have not tried extensive contour work, for I have not worked sufficiently long with it, but I would not hesitate to fill contour cavities of molars and bicuspids and large occlusal cavities.

*Dr. James Trumun.*—My microscopic examinations in this direction have led me to believe that it is extremely difficult to isolate a crystal of gold, but I have succeeded in repeated instances, and found it to be simply the usual form of crystal and not the aggregation we see. Some of the manufacturers of gold speak of the crystals of gold as being "fan-shaped." It is a great error and ought to be corrected.

I have had no experience with this gold. For a number of years I avoided all sponge golds of any character, for the simple reason that I was never able to get my edges satisfactory, owing to the sliding of the crystal, which gives the edges a spongy character. I do not think we take sufficiently into consideration the action of the crystals of any of the metals we use. In so-called rolled gold any variation in rolling will destroy the crystalline character, and we will not get cohesion. The same thing occurs with tin. I early abandoned rolled gold because of this defect in preparation.

*Dr. Gardiner.*—In speaking of crystals being larger, I did not mean to speak accurately. I should have said, "an apparent increase in the size of crystals;" but probably *coarseness of fibres* would be more correct. So far I have had no difficulty in obtaining good margins with this gold in the cavities in which I have used it, and I think that the gold, when it is thoroughly worked and condensed, is fully as hard as any other that I have used. It has the appearance of being hard, at least.

*Dr. Huey.*—Some of us will remember a form of sponge gold put on the market about twenty years ago, that brought distress to a good many, myself among others. I had to replace all operations I made with that gold, and I then vowed that I would never touch a sponge gold again. I was induced, however, to experiment with this new form of gold, but hesitated to risk it in places where there would be much leverage, fearful that it would break away. Dr. Truman's note of warning is very wise.

*Dr. Roberts.*—While we are on the gold subject, I would like to get the experience of some one who has used rolled platinized gold extensively as to the result after having it in the mouth for a length of time. There are some qualities about the filling, after it is finished, which are very pleasing, particularly in the front teeth, where the filling shows. It is much harder and is very frequently so nearly the color of the tooth that it is unnoticeable; but whether it will retain the same beautiful effect after it has been in the mouth for a number of years I do not know.

*Dr. Peters.*—I have not used this gold very much, but have tried it a few times, using shades Nos. 2 and 3, but did not get any of the results that Dr. Roberts speaks of. I think it is as noticeable as any other gold. It is certainly much harder. In any other way I do not see any improvement.

*Dr. Gardiner.*—I have been using rolled platinized gold extensively for the last few months, and I am very much pleased with the results, particularly in locations exposed to view. It is preferable to the pure gold in appearance, and its superior hardness fits it for extending the cutting edges of teeth. I have been doing some quite extensive operations; placing it beside some pure gold operations, and I think any one would agree that the platinized gold ones are far preferable in appearance. I do not fill the anchorages with it because it is stiffer; though it could be used in that way. I fill the anchorages with pure gold, because it is done more readily, and then add the rolled platinized gold to it. The gold should be annealed in the flame to a white heat to bring out both softness and cohesiveness. All that is necessary is to bring the surfaces into contact. It does not take more than ordinary force to weld it. The first work I ever saw done with it was a restoration of the cusp of an upper molar brought into hard wear. The edges were perfect, it was not pitted, and, in fact, it was an ideal operation in every way.

*Dr. Fellows.*—Some years ago I had a patient come to me to have a few teeth extracted; she had been wearing a plate for perhaps fifteen years. There were but one or two teeth to be removed, and afterwards I placed a plate in her mouth. In the course of a year or so I heard through one member of the family that she had been having a good deal of soreness about the mouth. When removing the teeth I noticed that there was an elevation in the palate running from the alveolar process towards the centre of the vault, and I took it to be an irregularity or malformation. When, a year later, I examined it I found that she had an impacted cuspid. It was impossible to get it out by ordinary means, but after a little cutting I was able to remove it entirely, and found that the enamel which was rubbed by the plate was very much resorbed and pitted. It seemed as though nature had attempted to remove the part causing irritation. I had an idea that it had not been resorbed until the plate was put in the mouth. I have the specimen in my pocket, if any one cares to look at it.

A second case is that of a patient who had trouble with a bicuspid tooth. The root was so badly affected with calculus that I thought it was best to extract it. I made an examination, and I found it was filled with gold almost to the end of the root. There evidently had been a good deal of irritation, and, in consequence, nature had attempted to resorb the root, the end of which was much pitted. The specimen is interesting in that evidently the operator had worked very carefully to fill the root with gold and had made a failure. I have never seen a root filled with gold that was filled perfectly, and I have seen a good many. This had been filled a good many years.

I had another case, some ten years or more ago, of a physician who had a tooth treated while attending college. A dental student put in a pellet of cotton with arsenic on it, and covered it over with gutta-percha. The gentleman did not return to the student, but was in practice some fifteen years when he came to my office, and when I opened into that tooth I found the pulp dead and the cotten intact, nor was there any harm caused by the arsenic he had placed there. I could not find any special odor. I merely bring these cases up for the purpose of showing how long cotton fibres will remain intact and how imperfect a gold root-filling is apt to be.

*Dr. Cryer.*—With reference to these canine teeth, the enamel is either decayed or resorbed. The imperfection may have taken place during the formation of that tooth, it not having originally the power to get into its position. I have extracted many of like character, where there have not been plates inserted in the mouth. In a specimen that was illustrated in the *Dental Cosmos*, there were thirteen teeth surrounding an impacted central incisor. In that case the central incisor was defective in the way in which this canine tooth is. I think it is more than likely that the artificial plate had nothing to do with causing the defect. It might cause the absorption of bone and gum-tissue and expose the root or tooth in that way, but I do not think it would affect the enamel.

Adjourned.

Otto E. Inglis,
*Editor Academy of Stomatology.*

